# A case of ulcerative tuberculous cellulitis in the setting of methotrexate-associated lymphoproliferative disorder

**DOI:** 10.1016/j.jdcr.2021.12.016

**Published:** 2022-01-06

**Authors:** Yohya Shigehara, Takahiro Mizuta, Sachie Kasami, Nanase Honda, Kota Shimada, Mikio Takamori, Yukihiko Katou, Yoshiko Mizukawa, Manabu Ohyama, Miyuki Kato

**Affiliations:** a Department of Dermatology, Tokyo Metropolitan Tama Medical Center, Tokyo, Japan; b Department of Rheumatic Diseases, Tokyo Metropolitan Tama Medical Center, Tokyo, Japan; c Department of Respiratory Medicine, Tokyo Metropolitan Tama Medical Center, Tokyo, Japan; d Department of Dermatology, Kyorin University Faculty of Medicine, Tokyo, Japan

**Keywords:** cutaneous tuberculosis, methotrexate-associated lymphoproliferative disorder, *Mycobacterium tuberculosis*, non-HIV immune reconstitution inflammatory syndrome, tuberculous cellulitis, CTB, cutaneous tuberculosis, IRIS, immune reconstitution inflammatory syndrome, MTX, methotrexate, MTX-LPD, methotrexate-associated lymphoproliferative disorder, Tac, tacrolimus, TB, tuberculosis

## Introduction

Cutaneous tuberculosis (CTB) can be classified into 2 major categories: true CTB and tuberculids. True CTB includes scrofuloderma, lupus vulgaris, tuberculous gumma, orificial tuberculosis (TB), and acute miliary TB. Tuberculids, including papulonecrotic tuberculid, lichen scrofulosorum, and Bazin erythema induratum, are an allergic reaction to *Mycobacterium tuberculosis.* Unlike with true CTB, organisms are not generally detected in tuberculids.

Tuberculous cellulitis is a rare form of CTB with cellulitis-like plaques, and it usually affects immunocompromised patients.^1^ Early recognition and treatment may prevent its progression but are often difficult due to overlapping features with other skin conditions.^2^

Several cases of atypical CTB have been reported in adults.^1^^,^^3^, ^4^, ^5^, ^6^, ^7^ We present a rare case of ulcerative tuberculous cellulitis in a patient with methotrexate-associated lymphoproliferative disorder (MTX-LPD.)

## Case report

**Fig 1:**
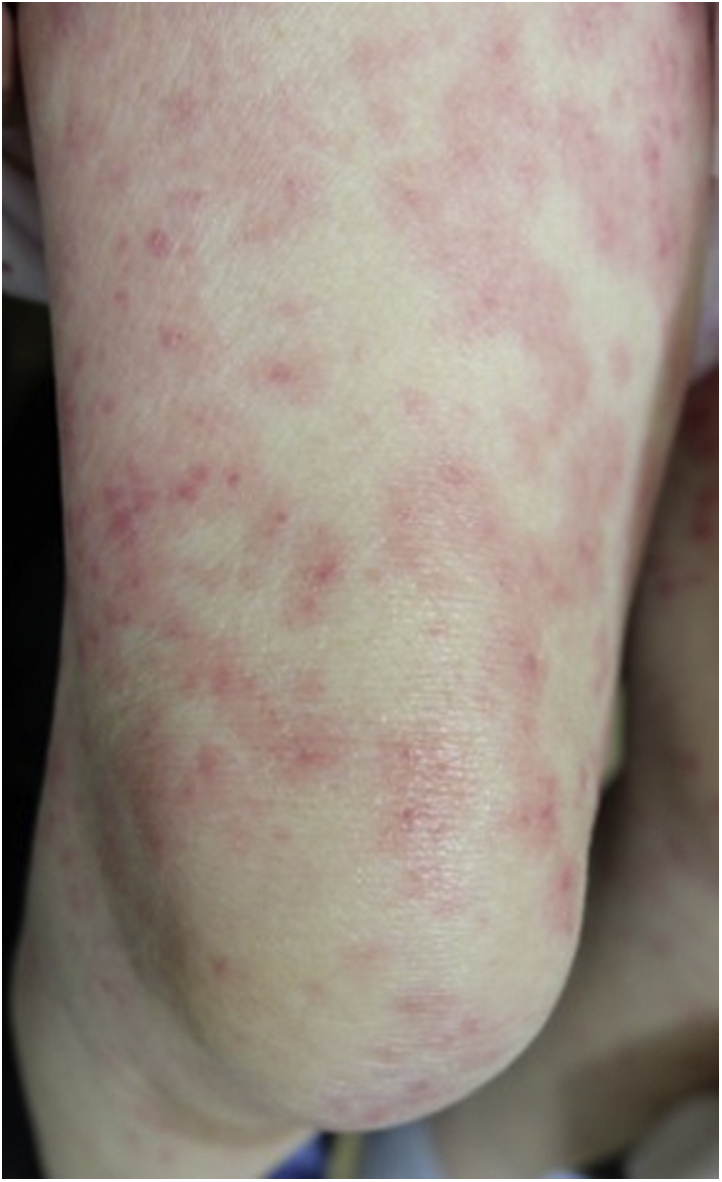
Erythema multiforme-like eruption. The patient presented with a skin eruption, characterized by a typical target lesion, on her extremities.

**Fig 2:**
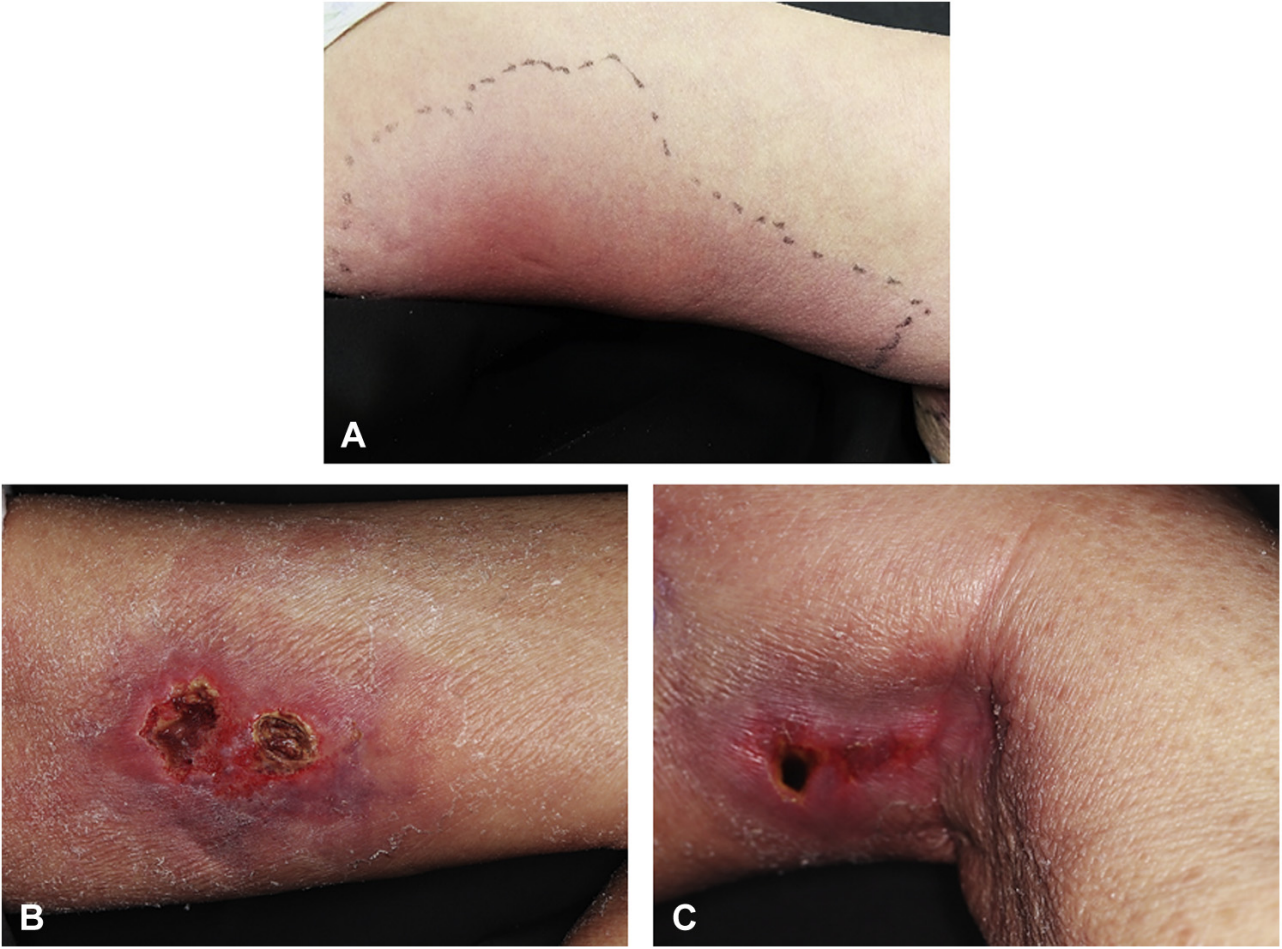
Ulcerative tuberculous cellulitis. **A**, Initial presentation showed painful indurated erythematous plaque on the medial side of the left thigh. **B**, Suture at the skin biopsy site on the medial side of the left thigh was compromised by an exudative ulcer. **C,** New ulcer on the back of the left knee.

An 85-year-old woman had been treated with methotrexate (MTX; 8 mg/week) and tacrolimus (2 mg/day) for rheumatoid arthritis for over a year. She had experienced tonsillar swelling 6 weeks before presentation. Her mother had died of TB several decades previously, but the patient had no TB history. Histopathology of a tonsillar biopsy revealed diffuse large B-cell lymphoma-type MTX-LPD and Epstein-Barr-virus–encoded small RNA in situ hybridization. The tonsillar swelling improved immediately following MTX and tacrolimus discontinuation. However, a week later, the patient developed an itchy erythema multiforme-like eruption on her extremities (Fig 1), which was treated with oral prednisolone, starting at 20 mg per day and gradually tapered. She developed a high fever (39.0 °C) and a painful, indurated erythematous plaque on the medial side of her left thigh (Fig 2, *A*).

Laboratory test results revealed an increased C-reactive protein level (11.14 mg/dL; reference range, ≤0.3 mg/dL). The cellulitis-like lesion did not improve after prednisolone withdrawal despite a 1-week course of intravenous antibiotics. A skin biopsy of the plaque on the patient’s left thigh led to dehiscence at the biopsy site, resulting in ulceration. A few days later, a new ulcer appeared on the medial side of the patient’s left thigh and at the back of her left knee (Fig 2, *B* and *C*).

**Fig 3:**
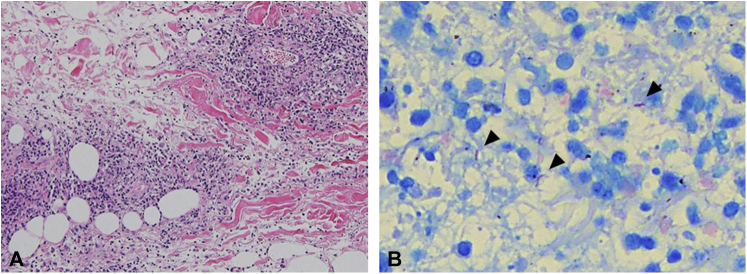
Histopathologic findings of the skin biopsy specimen performed for the indurated erythematous swelling on the patient’s medial side of the left thigh. **A**, Perivenous infiltration of numerous neutrophils with leukocytoclastic vasculitis and lobular panniculitis from the reticular dermis to the subcutis; caseous necrosis or epithelioid cell granuloma was not detected (Hematoxylin-eosin stain; original magnification: ×200.) **B**, Acid-fast bacilli were demonstrated through Ziehl-Neelsen staining (*arrowheads*) (Ziehl-Neelsen stain; original magnification: ×400.)

The skin biopsy revealed perivenous infiltration of numerous neutrophils with leukocytoclastic vasculitis and lobular panniculitis from the reticular dermis to the subcutis (Fig 3, *A*). No caseous necrosis or epithelioid cell granuloma was detected. However, Ziehl-Neelsen staining revealed acid-fast bacilli (Fig 3, *B*). Quantiferon-TB Gold Plus (QIAGEN) was positive for *M. tuberculosis*. *M. tuberculosis* was also detected on blood culture using Ogawa medium and in skin tissue using a polymerase chain reaction. Thus, ulcerative tuberculous cellulitis in the setting of MTX-LPD was diagnosed. Chest radiography and computed tomography from the neck to the pelvis revealed no tuberculous lesions.

Antituberculous therapy with isoniazid, rifampicin, and ethambutol was initiated 16 days after hospitalization, for 1 week, but was terminated because the patient developed a generalized drug eruption with severe itching. Pyrazinamide monotherapy was initiated 1 week later, once the symptoms had subsided. Immediately after restarting pyrazinamide, however, the patient developed a generalized drug eruption with severe itching. Therefore, pyrazinamide was discontinued. Levofloxacin, isoniazid, and rifampicin therapy was tried for several days. However, all 3 drugs were discontinued 42 days after admission due to repeated drug eruptions and progressive drug-induced renal injury. Both ulcers gradually worsened and were complicated by methicillin-resistant *Staphylococcus aureus* infection. The patient died 58 days after admission.

## Discussion

Tuberculous cellulitis presents with various cutaneous manifestations in immunosuppressed patients. To date, there have been 7 clinical reports of tuberculous cellulitis in adult patients, including our case (Table I).^1^^,^^3^, ^4^, ^5^, ^6^, ^7^ All patients were immunosuppressed. Six received steroid therapy, including 3 patients with collagen vascular disease,^4^, ^5^ 1 with diabetes,^1^ 1 with chronic hepatitis,^3^ and a renal transplant recipient.^7^ The extremities were affected in 6 of the 7 patients,^3^, ^4^, ^5^, ^6^, ^7^ and most developed concomitant bacterial superinfection. Several studies have reported severe tuberculous cellulitis causing erosions and ulcers and myositis, depending on the depth of invasion.^5^, ^6^, ^7^

**Table I:** Reported cases of tuberculous cellulitis in adult patients presenting with atypical cutaneous manifestations

Reference	Age (y)	Sex	Distribution	Clinical presentation	Associated diseases	Immunosuppressive therapy
Lee et al^1^	63	F	Abdomen	Erythematous swelling	Arthralgia, diabetes mellitus	Oral corticosteroids
Seyahi et al^3^	37	M	Left elbow, left calf and foot	Erythematous swelling	CKD, chronic hepatitis	mPSL 8 mg daily, AZA 100 mg daily
Kim et al^4^	47	F	Right axilla	Erythematous swelling	Dermatomyositis, gastric cancer	PSL 15 mg daily
Taguchi et al^5^	67	F	Right thumb	Erosion with maceration	SLE	Oral steroid therapy
Rabiei et al^6^	54	M	Left hand	Ulcer	Healthy	No medication
Muregesh Anand et al^7^	31	M	Right foot	Myositis	Renal transplant recipient	Tac 10 mg daily, MMF 360 mg daily, PSL 10 mg daily
Current case	85	F	Left thigh	Multiple ulcers	Rheumatoid arthritis, MTX-LPD	PSL 5 mg daily

*AZA*, Azathioprine; *CKD*, chronic kidney disease; *F,* female; *M,* male; *MMF*, mycophenolate mofetil; *mPSL*, methylprednisolone; *MTX-LPD*, methotrexate-associated lymphoproliferative disorder; *PSL*, prednisolone; *SLE*, systemic lupus erythematosus; *Tac*, tacrolimus; *y,* years.

Rapid polymerase chain reaction might be useful in cases of suspected tuberculous cellulitis, as its results enable early diagnosis. Although tuberculids such as erythema induratum are often positive on polymerase chain reaction tests, true CTB is confirmed when *M. tuberculosis* is detected by Ziehl-Neelsen staining or culture. All 3 tests were positive in our case; therefore, tuberculous cellulitis was diagnosed. Moreover, a broad diagnostic workup for mycobacterial and fungal infections is also important, because immunosuppressed patients often have other infections. Therefore, evaluation by dermatologists and infectious disease specialists is the best option for patients with cellulitis that is unresponsive to antibiotics.

Among the 7 reported cases of tuberculous cellulitis, only 3 patients had caseous necrosis or epithelioid cell granuloma on histology. The reason for the absence of caseous necrosis and epithelioid cell granuloma on histology in some cases is unclear. Steroid therapy may influence granuloma formation in patients with *M. tuberculosis* infection. Epstein-Barr virus and *M. tuberculosis* infections manifested over time in this immunocompromised patient, suggesting possible immune reconstitution inflammatory syndrome (IRIS). TB and CTB are common manifestations of IRIS in patients who test positive for HIV and undergo antiretroviral therapy.^8^^,^^9^ TB cases involving non-HIV IRIS have also been described recently.^10^ In this patient, the discontinuation of MTX and tacrolimus after MTX-LPD onset may have caused non-HIV IRIS and the development of ulcerative tuberculous cellulitis. We retrospectively speculate that the erythema multiforme-like eruption and the multiple drug eruptions were probably manifestations of non-HIV IRIS.

In conclusion, tuberculous cellulitis may be difficult to diagnose in immunocompromised patients. Physicians should consider the possibility of CTB when treating immunosuppressed patients with cellulitis that is unresponsive to antibiotic treatment.

## Conflicts of interest

None disclosed.

## References

[1] Lee N.H., Choi E.H., Lee W.S., Ahn S.K. (2000). Tuberculous cellulitis. Clin Exp Dermatol.

[2] Sakiyama M., Maeshima H., Chishiki M., Horinosono H., Kawakubo Y. (2016). Tuberculous cellulitis: diseases behind cellulitis like erythema. Cutis.

[3] Seyahi N., Apaydin S., Kahveci A., Mert A., Sariyar M., Erek E. (2005). Cellulitis as a manifestation of miliary tuberculosis in a renal transplant recipient. Transpl Infect Dis.

[4] Kim J.E., Ko J.Y., Bae S.C., Ro Y.S. (2011). Tuberculous cellulitis as a manifestation of miliary tuberculosis in a patient with malignancy-associated dermatomyositis. J Am Acad Dermatol.

[5] Taguchi R., Nakanishi T., Imanishi H., Ozawa T., Tsuruta D. (2015). A case of tuberculous cellulitis. Clin Med Insights Case Rep.

[6] Rabiei P., Hasanov M., Akhavan B., Aisenberg G.M. (2019). Tuberculous cellulitis in an immunocompetent patient. Proc (Bayl Univ Med Cent).

[7] Murugesh Anand S., Edwin Fernando M., Srinivasaprasad N.D., Sujit S., Thirumalvalavan K. (2020). Tuberculous myositis and cellulitis in a renal transplant recipient. Indian J Tuberc.

[8] Shelburne S.A., Montes M., Hamill R.J. (2006). Immune reconstitution inflammatory syndrome: more answers, more questions. J Antimicrob Chemother.

[9] Mann D., Sant’Anna F.M., Schmaltz C.A.S. (2018). Cutaneous tuberculosis and HIV infection at a referral centre in Rio de Janeiro, Brazil. Mem Inst Oswaldo Cruz.

[10] Sun H.Y., Singh N. (2009). Immune reconstitution inflammatory syndrome in non-HIV immunocompromised patients. Curr Opin Infect Dis.

